# Cultural Insiders and Graphic Stories to Promote Research Readiness Among the South Asian Community: A Focus on Purpose, Protection, and Participation

**DOI:** 10.3390/ijerph21101387

**Published:** 2024-10-19

**Authors:** Yatra N. Patel, Riya J. Patel, Lauren Bates, Susan Gertz, Susan Hershberger, Melinda Butsch Kovacic

**Affiliations:** 1 Cancer Research Scholars Program, The University of Cincinnati Cancer Center, Cincinnati, OH 45267, USA; pately4@mail.uc.edu (Y.N.P.); patel7rj@mail.uc.edu (R.J.P.); 2 Cincinnati Children’s Hospital Medical Center, Department of Pediatrics, College of Medicine, University of Cincinnati, Cincinnati, OH 45229, USA; lauren.bates@survivorjourneys.org; 3 Survivor Journeys, Longmeadow, MA 01116, USA; 4 Center for Chemistry Education, Department of Chemistry and Biochemistry, Miami University, Oxford, OH 45056, USA; gertzse@miamioh.edu (S.G.); hershbss@miamioh.edu (S.H.); 5 Department of Rehabilitation, Exercise, and Nutrition Sciences, the College of Allied Health Sciences, University of Cincinnati, Cincinnati, OH 45267, USA; 6 Office of Community Outreach and Engagement, The University of Cincinnati Cancer Center, Cincinnati, OH 45219, USA

**Keywords:** South Asians, community engagement, community outreach, graphic-style stories, research participation, cultural insiders

## Abstract

South Asians living in the United States are frequently underrepresented in health research. Their lack of participation limits the generalizability of research to them and keeps them from receiving the high-quality care and innovation that some studies may offer. “Research Ready” is a five-panel, community co-created graphic-style story that encourages discussion around the purpose of research, safety/protection while participating, and why diverse participation—including South Asians—improves study results and leads to more effective interventions/treatments. This study leveraged trained young adult “cultural insiders” to invite attendees of a Midwestern South Asian Cultural Festival to read the story aloud together as the characters in English or Hindi and used a decision guide to invite discussion. Post-discussion surveys (*N* = 104) were analyzed using descriptive statistics. Participants spanned from 10 to 79 years, with 42% < 18 years and more females (61%). Only 18.3% indicated having prior research participation. Adults 40+ years (60%) requested the story/discussion in Hindi, compared to 2.3% of adolescents and 6.7% of younger adults. After the discussion, participants indicated their willingness to consider participation, with most being open to participating in surveys/interviews (95.2%); only 52.9% would consider studies requiring the taking of medicines. Adolescents, females, and adults with higher education were more willing to participate in medication studies. Nearly all (97.1%) said they would feel safe participating in research, and 88.5% shared that the discussion would help them better decide about future participation. In conclusion, “Research Ready” discussions shared by cultural insiders effectively encourage South Asians to consider future research participation.

## 1. Introduction

Research conducted on homogenous populations can often lead to biased and misleading results, hindering the development of effective interventions and treatments for diverse populations [1]. This is particularly concerning in healthcare, where one-size-fits-all approaches often fail to address specific groups’ unique needs and experiences. Unfortunately, in the United States, research participation remains disproportionately low for populations like South Asians [2]. This lack of representation has far-reaching consequences, limiting the generalizability of research findings, perpetuating health inequities, and keeping individuals from receiving the high-quality care and innovation that some research may offer.

Asian Americans, compared to other racial and ethnic groups, have consistently trended to have lower research participation rates [2]. Therefore, understanding the specific barriers and facilitators within South Asian communities is crucial to designing effective strategies for recruitment and engagement. A scoping review by Quay et al. [3] identified disinterest, lack of education, logistical challenges or opportunity costs, and elements related to fear or inhibition as participant-related barriers. Themes of culture or language-related issues, logistical issues, issues related to study design, and lack of awareness were noted as researcher-related barriers impacting participation. The facilitating factors highlighted included seeing research as a route to better treatment or health, contributing to scientific knowledge or helping society, and having an obligation to their healthcare providers.

Mukherjea et al. (2018) [4] also published a case study series to expose barriers in recruiting South Asians within four diverse study designs that identified similar barriers. Based on their analysis, however, the paper also included strategies for recruiting South Asians into research. These included providing culturally appropriate presentations about relevant studies in the community, ideally within authentic partnerships with community organizations; leveraging respected leaders, including those of trusted community organizations, as messengers; and actively engaging cultural brokers (aka insiders) in the recruitment process. Their findings highlight the need for culturally tailored interventions that address these specific concerns and promote community engagement. Culturally tailored health education programming has been shown to help people make more informed choices, build self-efficacy, better engage with health information, and improve their health outcomes [5]. For decades, investigators who have partnered with community organizations and representatives (via participatory action research, community-based participatory research, etc.) have leveraged cultural insiders to inform their research and improve the recruitment and retention of minorities in clinical trials [6].

Within Greater Cincinnati, the Indian population forms one of the largest immigrant groups, boasting at least 16,854 foreign-born residents alone [7]. However, a lack of awareness and cultural barriers remain significant obstacles to their participation in research. To circumvent these challenges, the research described herein has leveraged a novel community education program that comprises a graphic-style story co-created with community representatives that encourages community discussions around the purpose of research, safety while participating, and why diverse participation—including South Asians —improves the generalizability of study results and leads to more effective interventions and treatments. Culturally tailored interventions require insight from the community itself.

Unlike most other outreach studies implemented to encourage recruitment into specific research studies, the community outreach program described was focused on building trust with the South Asian community by providing education about research participation without attempting to enroll participants into any particular study. The program leveraged two trained young adult “cultural insiders” to invite attendees of a Midwestern South Asian Cultural Festival to read a five-panel graphic-style story together and used a decision guide to further invite meaningful discussion around research participation. These young adult “cultural insiders” resided, worked, and practiced faith within the Indian community. Such embedded lay facilitators exert a greater impact on community education owing to increased trust and shared cultural context [8]. Benefits of leveraging cultural insiders include having a deeper understanding of the community’s culture and traditions and more easily building rapport with program participants. Cultural insiders often have a deeper understanding of social characteristics, exclusive knowledge about the community, and cultural competency, which all facilitate effective engagement and communication. As such, cultural insiders may also gain access to community participants more easily than outsiders. Once access is gained, social characteristics, exclusive knowledge about the community, and cultural competency all facilitate effective communication.

For this study, rather than offering education via a formal presentation, the insiders were trained to facilitate the reading of a short graphic-style story aloud together as the characters in either English or Hindi. Content delivery using stories is an engaging approach to sharing health and science information. It also accommodates individuals of various ages and literacy levels [9]. The use of colorful characters ensures that people from all backgrounds feel included and can resonate with the story. The training helped the insiders to use the story to more easily convey important facts and allow those facts to become a starting place for discussion. As the trained insiders facilitate the discussion non-judgmentally by asking and answering questions, they help participants come to their own conclusions about health research participation without feeling pressured to participate.

By understanding the specific needs of South Asians, cultural insiders can help research-hesitant community members to bridge the knowledge gap about research participation and improve their “research readiness” via the easy-to-use Research Ready story. The result is a more inclusive research environment that benefits both the South Asian and scientific communities. Indeed, by increasing diverse research participation, healthcare solutions that are more effective for a broader range of people can be developed. Research considering different perspectives is more likely to identify potential biases or unintended consequences.

## 2. Materials and Methods

### 2.1. Creation of Graphic-Style Stories with Community Partners

**Figure 1:**
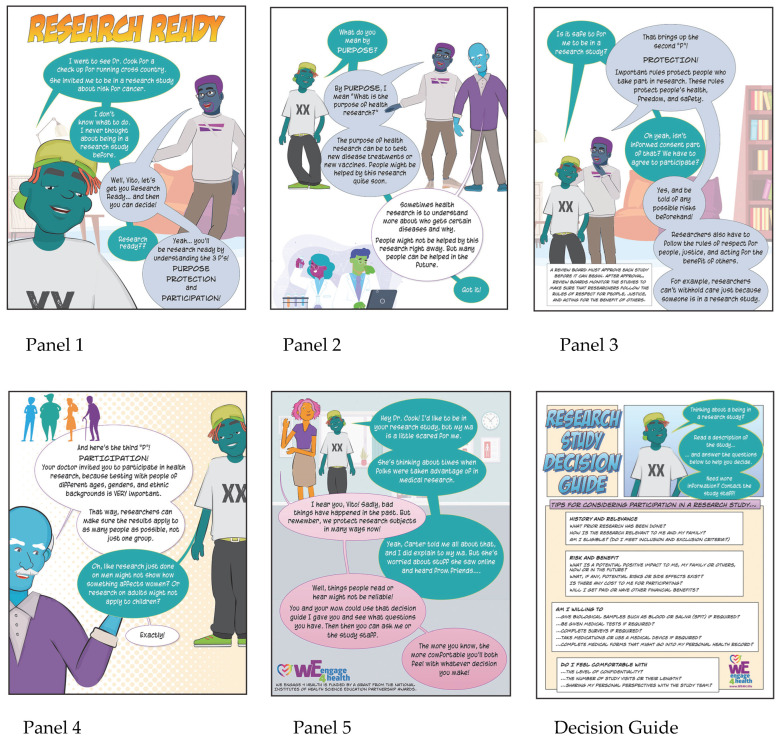
Five-panel Research Ready story and the accompanying Research Study Decision Guide.

We Engage 4 Health (WE4H) is a 20+ member interdisciplinary community–academic partnership involving the University of Cincinnati (UC), Cincinnati Children’s Hospital Medical Center (CCHMC), Miami University, and Seven Hills Neighborhood Houses in Cincinnati’s West End. WE4H uses graphic-style stories that were iteratively co-created with community members. While all our community representatives considered themselves African American, typically 2–4 members of WE4H (primarily interns) considered themselves South Asian at any given time. One of their primary partners is the West End Community Research Advisory Board (WE C-RAB). WE C-RAB is an institutionally supported board that has been working together since 2017 to provide a community perspective to health researchers from UC and CCHMC and support health promotion efforts in the West End. In 2020, members of WE C-RAB expressed the desire to “be able to better talk to their family, friends, and neighbors about research and its potential impact on their health”. A 23-panel “Research Ready” story was co-created through numerous iterations with board members. The story focused on the purpose of health research, how people are protected from harm while participating, and why diverse participation is needed to produce useful results. After that, WE4H created the Research Study Review Guide (Figure 1, Decision Guide) to help people consider the history and relevance of research, its risks, and benefits, what they were and were not willing to do as participants, and what their comfort level was with the specific research requirements. Early testing of the 23-panel story revealed it as a valuable resource in the classroom and personal settings but too long for quick encounters at health fairs and community events.

For this reason, a 5-panel version of “Research Ready” was developed (Figure 1, Panels 1–5). The new shorter story was designed to enable a rich discussion with more interactive questioning in less time. Furthermore, translating the story into Hindi ensured its accessibility to a broader audience. A “Research Ready” storybook, including both the longer and short stories as well as the Research Study Decision Guide, was printed for story discussion participants to take home with them to learn more and to share with their families and friends. A digital version of the “Research Ready” storybook is available through the Open Science Foundation website [10].

### 2.2. Training Community Research Advocates for the Research Ready Outreach Program

The Community Research Advocate (CRA) program trains volunteers to effectively inform their community members about research participation. The program seeks CRA candidates who are passionate about community health and research, adept at connecting with people, organizing meetings, and managing time effectively while also being available to collaborate with co-CRAs on Research Ready discussions. This 2-h training included a virtual PowerPoint presentation that covered the 5 E’s—Engage (introduce yourself and let others introduce themselves); Explore (read the story aloud as the characters with participants); Explain (facilitate a non-judgmental discussion and discuss the 3 P’s); Expand (use the Research Study Decision Guide and a featured study to practice decision-making about participation); and Evaluate (invite participants to voluntarily complete a post-discussion survey, administer the survey, and log their discussions thereafter). The training focused on helping CRAs understand the concepts of being “research ready”, practicing facilitation of non-judgmental discussions using active listening skills and the APPLE technique (ask a question, pause to allow consideration of a response, pick a volunteer to answer, listen to the response, elaborate on the response reflecting back what was said). CRAs were instructed not to judge participants for their feelings and beliefs related to participation and to instead look for participants’ underlying concerns (e.g., mistrust, issues with safety, etc.). Rather than correcting participants directly, CRAs were instructed to allow the reading of the story and discussion to reveal facts that might alleviate their issues.

The Research Ready Checklist was created from these training materials to outline the CRAs’ steps for each discussion and keep them from accidentally skipping steps during their outreach. CRAs could also use it to track their fidelity to the program’s protocol, and CRAs were asked to attach it to the CRA Log associated with each event (Table 1). CRAs were also instructed to review all post-discussion surveys collected, ask participants to complete missing responses if willing, and thank them for completing the survey. CRAs were given access to a digital CRA Outreach Guide, which included the training slides and was presented as a resource for the future. These materials are available through the Open Science Foundation website [10]. Recognizing the significance of cultural nuances, two South Asian cultural insiders, fluent in Gujarati, Hindi, and English, were trained as CRAs, bridging language barriers and ensuring the information resonated with South Asian community members. Following their training, trainees completed the Research Ready Knowledge Check online, designed in REDCap to assess their story comprehension and facilitation skills. Both insiders responded to all quiz questions correctly.

### 2.3. Material Preparation and Program Engagement

The trained CRAs had previously attended a 2-day local cultural fair focused on food and fun attended mainly by South Asians. They felt that people would be open to being approached during the event. Capitalizing on this insider knowledge, they chose the event as the setting for their outreach. As trusted organizations hosted the festival, and all “vendors” were vetted by organizers, it was hypothesized that for this program, participants would likely feel greater safety when approached by the CRAs.

To facilitate the effective dissemination of information about research participation at the 2-day event, the CRAs prepared a comprehensive set of materials. They printed copies of the Research Ready graphic stories in English and Hindi. The CRAs also printed post-discussion surveys in both languages. They also gathered examples of current and applicable research study opportunities in the Cincinnati area, highlighting opportunities for participation in relevant research. These cards included key information about the study and a QR code linked to a REDCap survey for those interested in learning more. Furthermore, additional materials such as hand sanitizer, fidget toys, WE4H logo water bottles, and other small tokens of appreciation were prepared for distribution to attract and thank community members for their participation.

Equipped with these prepared materials, the CRAs attended this two-day annual event known to attract a large South Asian community. The CRAs primarily sought out people traveling in groups of 4–7 to read the Research Ready story out loud together. Maintaining the highest ethical standards, CRAs ensured complete voluntariness in all program activities. The CRAs obtained verbal consent from everyone participating in the discussions and surveys. After briefly introducing the Research Ready program and its mission, CRAs tailored their approach to each participant. Speaking in either English, Gujarati, or Hindi to accommodate individual preferences, they distributed copies of the language-specific stories and facilitated interactive read-aloud sessions. Each participant was assigned a story character and encouraged to embody their perspective throughout the discussion. The CRAs offered optional post-discussion surveys in English and Hindi to gather valuable insights and inform future outreach efforts. Upon request, CRAs provided information regarding current research study opportunities in Cincinnati.

### 2.4. Post-Discussion Survey

There were eight questions asking about participants’ demographics, including age, race, gender, language, and education level.

Two story-related comprehension questions were included to assess participants’ understanding of the key themes highlighted in the Research Ready graphic story (purpose, protection, and participation) and the definition of research readiness. A third question asked if they liked using a comic-style story to learn about research.

The next two questions asked participants about their prior research participation and if they had ever considered participating in research before their discussion. Response choices included yes, no, or I do not know. Another four questions were asked about their willingness to participate in varying types of research in the future, including completing surveys, contributing biological samples, undergoing study-related medical tests, or taking medication. These questions were similar to those used by Liu et al. [2]. Response choices included ”I would do”, ”I would consider doing”, “I would not do”. There were three questions exploring participants’ feelings of safety and decision-making. These questions asked participants to respond via a four-point Likert scale (strongly agree, agree, disagree, and strongly disagree).

The final six questions explored the appeal of potential research incentives. Participants were presented with a list of possible benefits and asked to select the ones they found most enticing. This approach aimed to identify the factors that motivate involvement, potentially revealing intrinsic, extrinsic, or a combination of factors.

The Research Ready evaluation protocol was reviewed by the UC Institutional Review Board. It was deemed “Exempt from Review” under Federal Regulation 45CFR46 (IRB Protocol #2021-1088).

### 2.5. Data Analysis

After the event, the CRAs manually entered completed surveys into REDCap. Data summaries were first drafted in Excel. The summaries included the percentages of participants for each response in the larger group and by age, gender, and education level. For the willingness-to-participate questions, participants responding, ”I would consider” and ”I would do” were compared to those who responded “I would not do”. For the safety and decision-making questions, those choosing “strongly agree” or “agree” were compared to those who “disagreed” or “strongly disagreed”. SPSS (version 29) was used to confirm the frequencies reported by Excel and identify statistically significant differences at the 0.05 confidence level using Chi-squared tests; 3 × 2 Fisher exact tests were performed using Vassarstats (http://vassarstats.net/fisher2x3.html (accessed on 11 October 2024)).

## 3. Results

Discussion participants were primarily South Asian (88%) and spanned from age 10 to 79, with 42.3% being less than 18 years old (Table 2). There were more female (61%) participants than male. Among adult participants, 36.7% had a high school diploma, while 61.7% had a bachelor’s or master’s degree or more. Slightly more than 20% of participants said that Hindi was their primary language. Only 18.3% indicated having previously participated in research. Prior participation increased by age, with 13.6% of children and adolescents, 16.7% of adults 18–39, and 25.7% of those age 40 and older. In total, 43.5% of participants had considered participating in research prior to their Research Ready discussions.

Interestingly, 60% of adults 40 and older requested to read and discuss the story in Hindi, compared to 2.3% of adolescents and 6.7% of younger adults. These participants also completed the post-discussion surveys in Hindi. The post-discussion survey included two questions to ascertain knowledge gained from the discussion; 95.2% of respondents understood the main story points—purpose, protection, and participation—and 97.1% understood the meaning of “Research Ready”.

After the discussion, nearly all participants (97.1%) said they would feel safe participating in research. However, when asked about their willingness to consider research participation, most were open to participating in surveys/interviews (95.2%), while only 52.9% would consider studies requiring taking medicines (Table 3).

There were no statistically significant group differences by age, gender, or education level. However, younger participants tended to be more willing to participate in each type of research than adults, and females were more willing than males. Participants with less education tended to be more willing to participate in studies collecting biological samples or completing medical tests. Considering all participants, just more than half indicated they would participate in medication/treatment-type studies regardless of their level of education; however, looking at adult participants only, those with higher education tended to be more willing to consider participation versus those with a high school diploma or less (54.1% vs. 39.1%, *p* = 0.26).

More than 63.5% of participants indicated they liked learning about research participation using a graphic-style story, and 88.5% shared that the Research Ready discussion would help them better decide about future participation in research.

Financial incentives emerged as the strongest motivator, with two-thirds of participants finding them appealing (Table 4). Interestingly, this desire for financial incentives significantly increased with age, with 80% of adults over age 40 valuing them (*p* = 0.0099). Conversely, receiving personal results from the study was particularly attractive to younger participants (nearly 80% of adolescents and teens) (*p* = 0001). Notably, ease of participation held consistent importance across all age groups, with roughly half of participants prioritizing it (*p* = 0.97). Learning or improving one’s own health was chosen significantly less by older adults than the other groups (*p* = 0.002). Considering the entire group, a provider or family recommendation held the least sway, with only 36.5% of participants finding it motivating. Interestingly, this influence seemed to significantly wane with age (*p* = 0.016).

Males tended to prefer receiving incentives 73.7% and females preferred receiving their personal results, though the differences were not statistically different. Significantly more females than males wished to make a community impact and appreciated having their provider or family member recommend a study (*p* = 0.0073 and 0.038, respectively).

Significant differences were also observed by education level. Those with higher levels of education cared most about personal results as well as learning something new to improve their health (*p* = 0.04 and 0.0003, respectively). Very few participants with a high school education or less desired a doctor or family recommendation (13.5% vs. 49.3%, *p* = 0.0003).

## 4. Discussion

South Asians are currently an underrepresented group in research. This study aimed to evaluate an outreach program designed to equip South Asian visitors at a Midwestern cultural music festival with the skills to consider and decide about future research participation through a more culturally aware, engaging approach. Indeed, two trained young adult CRAs serving as cultural insiders in the community utilized a graphic-style story read aloud together as the characters preempted brief but meaningful discussions about the purpose of research, how participants are protected from harm, and the need for diverse participation to improve the generalizability of the research results to people like them. Our analysis of post-discussion surveys reveals that this innovative approach significantly impacted participants’ understanding and openness to consider research participation and decision-making.

A wide age range of South Asians (10 to 79) participated in the outreach program and completed a post-discussion survey. More than two-thirds were female. Previous research suggests that females are often more likely to participate in research when approached by female researchers [4]. In this case, both cultural insiders were female, potentially contributing to this observation. Not surprisingly, adult participants with higher levels of education were more likely to have had prior research participation (*p* = 0.046). Others have reported that those with higher levels of education are more likely to be willing to participate in clinical research than those with lower education levels [11,12]. It has been well-established that higher education often leads to better health literacy and willingness to navigate the complexities of clinical research, including balancing the potential risks and benefits [11]; however, after participating in a Research Ready discussion, there were no significant differences in willingness to participate regardless of education level after participating in a Research Ready discussion. Notably, adults’ willingness to participate in medicine/treatment studies favored the more educated (54.1% vs. 39.1%, *p* = 0.26).

There was a positive trend between age and language preferences among the South Asian participants. Not surprisingly, only 2.3% of participants aged 10 to 17 preferred to read Research Ready in Hindi, whereas 6.7% of the 18 to 40 age group and 60% of the 40 plus age group favored Hindi (*p* < 0.001). Other studies have also found similar results, particularly in first-generation immigrants and older adults [13,14]. Among the South Asian participants in this study, it is possible that the older adults were more likely to have immigrated to the United States and, consequently, retain a stronger connection to their heritage languages. At the same time, adolescents were more likely to have grown up in the United States and, therefore, be more comfortable reading the story in English. This underscores the importance of translating both outreach and study materials into different languages while simultaneously considering cultural realities when designing outreach and research strategies to ensure inclusivity and maximize participation across age groups. Previous studies have found that the translation of materials alone can be inadequate [15].

Two questions were included in the post-discussion survey to assess basic knowledge of the three P’s (purpose, protection, and participation) and the definition of the term “research ready” as defined in the story. Most participants demonstrated exceptional knowledge retention, with 95.2% accuracy and 97.1% responding correctly, respectively, showing that using a graphic-style story is helpful in improving knowledge. Furthermore, 63.5% of participants indicated they liked learning about research participation using a graphic-style story. This finding did not vary by age. While others have reported that stories can benefit all learning types, stories are especially useful in working with those with limited health literacy, those typically underserved by traditional health communication approaches, as well as experiential and global learners who might benefit from a more experiential learning experience [16]. Research in experiential learning suggests that embodied learning leads to stronger memory consolidation and knowledge retention [17].

A critical goal of our research was to examine whether brief discussions following reading Research Ready aloud together as the characters empowered participants to make informed decisions about research participation or not. A little over 93% of participants reported feeling more confident considering future research opportunities after their Research Ready discussion, and 97% indicated they felt safe within the research environment after the discussion. This finding underscores the transformative power of culturally responsive community outreach in shaping informed decision-making about research participation. Interestingly, 42.3% of participants admitted never having contemplated research participation. This study population represents a previously untapped pool of potential research subjects.

Remarkably, after participating in a Research Ready discussion and receiving the Research Study Decision Guide, 95.2% of participants expressed willingness to complete surveys or participate in interviews, highlighting a general overall comfort with this relatively non-invasive research (Table 3). This preference for surveys held true across all age groups. However, as the potential invasiveness of research increased, willingness to participate diminished. In the end, only 52.9% reported a willingness to consider studies requiring taking medicines, such as clinical trials. This inverse trend is consistent with other studies, including those of diverse study populations [18]. Liu et al. [2] reported that 75.6% of their 485 Asian participants agreed to answer health-related survey questions, 61.7% were open to giving samples, 38.6% would undergo medical tests that require overnight stay at the hospital, and 30.5% were willing to take medications for research purposes. Unlike our study, their survey subjects were not given language options other than English before completing their survey [2]. They also were also not provided with any education about research before participating. Using the Research Study Decision Guide, our participants were given questions they could use to explore their values and with which to ask study staff questions that might help them to make better decisions.

The five-panel Research Ready story was developed to introduce and discuss the concept of human subjects research to community members who were relatively unfamiliar with research and provide them with skills to evaluate future opportunities. Considering the Theory of Change, the goal of Research Ready was not necessarily to convince people to participate in trials-based research, nor were they being actively recruited to any one study. Rather, the purpose of Research Ready was to move participants along the continuum of change from “pre-contemplation” to “contemplation” or from “preparation to “action” [19]. Indeed, for some, willingness to participate in surveys and interview-based research could be a first step in helping people to consider participation in studies that require subjects to provide biological samples or complete medical testing. If a person is open to those types of research and, upon participating, has a good experience, they may feel safer and more willing to participate in clinical trials if invited in the future.

Co-creation, co-design, and co-production with community representatives are more commonly used to address public health challenges [20]. The Research Ready—CRA program was co-created with community representatives for the community as part of a community-academic partnership. Far fewer research outreach programs published in the literature were co-created with community representatives, and even fewer are unattached from research recruitment efforts. Offering such low-pressure education programs could benefit institutions wanting to build trust with their larger communities. Indeed, scribe notes from the discussion at two “Health Equity through Diversity” webinars were thematically analyzed and described in a recent publication [21]. Importantly, participants noted that innovative, community-engaged, co-designed solutions are essential to addressing clinical trial diversity. In fact, in 2021, the National Cancer Institute issued guidelines for Cancer Center Support Grants specifying that community outreach and engagement experts be appointed to work with their Clinical Trials Offices to “facilitate accrual to clinical trials from the catchment area” as well as provide appropriate outreach efforts in the local communities [22]. Ideally, outreach should begin within vulnerable communities and not only when patients are asked to participate in research, particularly if the study invitation is being made during the stressful period shortly after a cancer diagnosis, when overwhelming the patient is likely to occur.

Our study also revealed age, gender, and education level differences in preferences for research incentives (Table 4). While older participants prioritized tangible rewards, younger participants placed greater value on understanding their personal health and receiving their results. Additionally, younger participants showed a heightened concern about the research’s impact on their community. Furthermore, older adults were less likely to require a doctor or family recommendation when participating in research. Herera et al. reported that older adults often highly valued their autonomy and independence, usually making their own decisions about research participation, even if it conflicts with recommendations [23]. A systematic review by Gaffney and Hamiduzzaman suggests that older adults might have trust issues with healthcare systems and research institutions alike [24]. While not statistically significant, our study found that 74% of men preferred to receive incentives compared to only 63% of women. Others have reported similar sex preferences [25]. Knowing these nuanced preferences allows researchers to tailor recruitment strategies, incentives, and communication styles to attract and retain participants from various age groups and demographic backgrounds.

While Research Ready is not likely to help South Asians overcome all of the barriers related to research participation identified by Quay et al. [3], it may increase their interest in considering research participation, diminish their fears or inhibitions—by increasing their knowledge of both its purpose and how people are protected from harm—and improve their awareness of the different kinds of potentially beneficial research available to them. Further, as other South Asians led the discussions, they might feel safer and more open to the “research ready” concepts shared. Indeed, “cultural brokering programs” have been used to help vulnerable groups to navigate healthcare for decades [26].

An essential limitation of this study is the absence of a pre-discussion survey. Without such a survey, our study team could not directly determine, for example, changes in feelings of safety or willingness to participate in future research. The lack of a pre-discussion survey was a deliberate choice for two reasons. First, there was a concern that asking attendees at a social event like a cultural festival to complete a survey before participating might discourage participation in the discussion. Second, a pre-discussion survey would not be typical in a real-world outreach program, potentially limiting the generalizability of our findings. Nonetheless, our post-discussion survey was able to ascertain participants’ history of research participation (as well as whether they had considered participating or not). This study’s findings demonstrate that most had never even considered participating in research before participating in the outreach activity.

Another possible limitation is that far fewer South Asians indicated a willingness to participate in medication/treatment studies after the discussion. This was not surprising to us, given the program is based on a singular and relatively brief discussion. Ongoing research is exploring the impact of a longer and more detailed version of Research Ready as well as a revised version called “Becoming Research Ready” (whose main character is an adult cancer survivor) offered in group settings. Additionally, we are examining the utility of sharing examples of different types of research studies during Research Ready discussions, including tangible examples of trials that are more likely to have direct benefits without considerable risk of harm, which could be beneficial.

Finally, while more participants indicated a willingness to participate after their Research Ready discussion, it is still being determined whether this will translate into actual research participation. Participants must be followed up over time to determine the program’s impact on research participation. Future longitudinal studies are required to measure research participation rates after a Research Ready discussion.

## 5. Conclusions

In conclusion, “Research Ready” discussions shared by cultural insiders effectively encourage South Asians to consider future research participation. Establishing trust through partnerships with community organizations and cultural ambassadors is a viable approach to creating outreach opportunities for greater diversity in study populations. Further, partnerships with schools, universities, and community organizations can facilitate reaching younger generations and encouraging movement along the Theory of Change continuum. Through active outreach about research participation with and in communities without the pressure of an impending ask to enroll in a study, greater equity in research participation can be achieved.

## Figures and Tables

**Table 1:** WE4H Research Ready discussion plan.

Check	#	Action
	1	Introduce yourself. Share where you’re from and a little bit about yourself.
	2	Explain that you’re going to start off with a very quick 4-question pre-discussion survey *. Explain that their participation is voluntary but appreciated. Collect surveys, making sure that all questions are completed and an identifier is on each.
	3	Introduce the concept of “research readiness”. Briefly go over the 3 P’s.
	4	Introduce the concept of “research” focusing on Human Subjects Research.
	5	Explain how the comic-style story will be used to initiate a meaningful discussion about research participation.
	6	Introduce the story characters and ask for volunteers to read each character. Read the story aloud as the characters with your participants.
	7	Ask our participants the following questions. After that, respond to the questions and discuss.
		Did you learn anything new? Did anything surprise you?
		Do you have any questions?
	8	Next, introduce the featured study and ask participants to review the study documents.
	9	Direct the participants to the Research Study Decision Guide and ask them to use it to evaluate the featured study. You can read the Guide with them to prompt consideration.
	10	Ask the following question: Considering what we read about Vito, would you expect Vito to participate in this study? Why or why not? Discuss.
	11	Invite those who feel comfortable doing so to share if they would feel comfortable participating, and ask them to explain why or why not. Then, invite further discussion and questions.
	12	Be willing to discuss types of research opportunities, including survey and focus group research, educational-type research, research collecting biological samples, and those asking you to get medical testing. Then, discuss clinical trials and medication-type research. Explain—some potential benefits of each.
	13	If you are targeting an adolescent group, discuss being a researcher as a career. Use yourself as an example to explain some of the decisions you made. Invite further questions/discussion about research as a career and education needed.
	14	Ask them to complete the post-discussion survey. Explain that the survey is voluntary but appreciated for helping us evaluate the Research Ready Program. Share that by completing the survey, they are consenting to participate. Collect surveys, making sure that all questions are completed and that an identifier is on each.
	15	Thank them for participating, and offer them incentives or giveaways as available/appropriate for those submitting completed surveys. Log your discussion in the online Research Ready Log.

* Pre-discussion survey added for select groups, not typically used for community health events.

**Table 2:** Population characteristics (*N* = 104).

Characteristic	%
Age (Years)	
10–17	42.3
18–40	28.9
41+	28.9
Race	
Asian	88.5
White	6.7
Black	1.9
Prefer Not to Answer	2.9
Gender	
Female	61.5
Male	36.5
Prefer Not to Answer	1.9
Primary Language	
English	79.8
Hindi	20.2
Education Among Adults	
<high school/in progress	1.0
High school diploma	21.2
2-year college/vocational degree	4.8
4-year college/vocational degree	30.0
Masters/professional/doctorate+	4.8
Past Research Experience	
Yes	18.3
No	81.7

**Table 3:** Willingness to consider participation in research (%).

Population/Subgroup	N	Surveys	Samples	Medical Tests	Medicines/Treatments
All Participants		95.2%	79.8%	70.2%	52.9%
By Age (*p*-value *)		0.39	0.53	0.38	0.54
10–17 years	44	97.7%	84.1%	77.3%	59.1%
18–40 years	30	90.0%	73.3%	66.7%	50.0%
41+ years	30	96.7%	80.0%	63.3%	46.7%
By Gender (*p*-value *)		1.0	0.55	0.28	0.26
Female	64	95.3%	81.3%	73.4%	56.3%
Male	38	94.7%	76.3%	63.2%	44.7%
By Education Level (*p*-value *)		0.83	0.44	0.078	0.86
High school diploma or less	67	95.5%	82.1%	76.1%	52.2%
Beyond high school diploma	37	94.6%	75.7%	59.5%	54.1%

* *p*-values based on Fisher’s exact test; two participants chose not to share their gender and are not included.

**Table 4:** Preferences for participant incentives.

Population/Subgroup	*N*	Incentives	Personal Results	Quick and Easy	Learn/Improve Health	Research Impacts Community/Others	Doctor/Family Recommendation
All Participants	104	66.3%	64.4%	55.8%	51.0%	44.2%	36.5%
By Age (*p*-value *)		0.0099	0.0001	0.97	0.0002	0.062	0.016
10–17 years	44	50.0%	79.6%	52.3%	58.6%	56.8%	52.3%
18–40 years	30	76.7%	73.3%	50.0%	25.9%	40.0%	26.7%
41+ years	30	80.0%	33.3%	50.0%	15.5%	30.0%	23.3%
By Gender (*p*-value*)		0.24	0.35	0.68	0.11	0.0073	0.038
Female	64	62.5%	67.2%	48.4%	60.9%	53.1%	43.8%
Male	38	73.7%	57.9%	52.6%	44.7%	26.3%	23.7%
By Education Level (*p*-value *)		0.53	0.04	0.64	0.0003	0.16	0.0003
High school diploma or less	67	70.3%	51.4%	54.1%	32.4%	35.1%	13.5%
Beyond high school diploma	37	64.2%	71.6%	49.3%	68.7%	49.3%	49.3%

* *p*-values based on Chi-square tests; two participants chose not to share their gender and are not included.

## Data Availability

The datasets generated and analyzed during this study are available from the corresponding authors on reasonable request or by accessing the Open Science Foundation DOI 10.17605/OSF.IO/M2VFJ.
